# P4HTM: A Novel Downstream Target of GATA3 in Breast Cancer

**DOI:** 10.21203/rs.3.rs-2622989/v1

**Published:** 2023-02-28

**Authors:** Sarah C. DiDonna, Aerica Nagornyuk, Neeta Adhikari, Mamoru Takada, Motoki Takaku

**Affiliations:** University of North Dakota School of Medicine and Health Sciences; University of North Dakota School of Medicine and Health Sciences; University of North Dakota School of Medicine and Health Sciences; Chiba University: Chiba Daigaku; University of North Dakota School of Medicine and Health Sciences

**Keywords:** P4HTM, GATA3, breast cancer

## Abstract

Breast cancer continues to be a major cause of death among women. The GATA3 gene is often overexpressed in breast cancer and is widely used to support a diagnosis. However, lower expression of GATA3 has been linked to poorer prognosis along with frequent gene mutations. Therefore, the role of GATA3 in breast cancer appears to be context specific. This study aims to identify a new downstream target of GATA3 to better understand its regulatory network. Clinical data analysis identified the prolyl 4-hydroxylase transmembrane protein (P4HTM) as one of the most highly co-expressed genes with GATA3. Immunohistochemical staining of breast tumors confirms co-expression between GATA3 and P4HTM at the protein level. Similar to GATA3, P4HTM expression levels are linked to patient prognosis, with lower levels indicating poorer survival. Genomics data found that GATA3 binds to the P4HTM locus, and that ectopic expression of GATA3 in basal breast cancer cells increases the P4HTM transcript level. These results collectively suggest that P4HTM is a novel downstream target of GATA3 in breast cancer and is involved in tumor progression.

## Introduction

Breast cancer is the most common and lethal type of cancer in women worldwide. Although extensive research in breast cancer has identified many important biomarkers such as ESR1, PGR, and HER2 that can guide patient treatment procedures, acquisition of hormone therapy resistance and treatment for metastatic breast cancer are still common issues (1). One such biomarker, GATA3, is a zinc-finger transcription factor that is important for normal mammary gland development and breast tumor development (2, 3). GATA3 is frequently overexpressed in luminal subtype of breast tumors, but its expression level positively correlates with breast cancer patient survival (4). Additionally, large patient cohort studies such as TCGA and METABRIC identified GATA3 as one of the most frequently mutated genes in breast cancer, and more than 10% of breast tumors carry GATA3 mutations (4–7). Recent studies from our group and others demonstrated that at least some of the mutations stimulate tumor growth by reprogramming the transcription landscape (4, 8–12). Consistently, genetic analysis in metastatic breast tumors revealed increased frequency of GATA3 mutations (13). However, clinical data suggest that breast tumors harboring GATA3 mutations exhibit a better prognosis in general, including higher patient survival rates (4). Therefore, the roles of GATA3 appear to be complex and likely context (e.g., breast cancer subtypes) specific (14).

Several key GATA3 downstream pathways have been discovered by in vitro studies. in MDA-MB-231 basal breast cancer cells, GATA3 has been shown to induce mesenchymal-to-epithelial transition by acting as a pioneer transcription factor, and the reprogrammed MDA-MB-231 cells become less metastatic (15, 16). in this cell reprogramming process, GATA3 binds to inactive epithelial genes and activates expression by converting closed chromatin to open chromatin (17). in luminal breast cancer cells, such as T47D and MCF7 cells, GATA3 works with Estrogen Receptor alpha (ER-a) and FOXA1, and this transcription factor network is essential for shaping and maintaining epithelial and luminal phenotypes (11, 18, 19). However, genomic data indicate that GATA3 binds to over 10,000 loci in these breast cancer cells, and that altering GATA3 expression results in thousands of transcription alterations (4, 17, 20). GATA3 must have many more direct targets, which could be important for breast cancer characterization.

In this study, we sought to identify a novel GATA3 target that is important for breast cancer progression. In the largest breast cancer cohort, P4HTM mRNA expression was found to be most significantly correlated with GATA3 expression level. Similar to GATA3, P4HTM is up-regulated in luminal subtypes and down-regulated in basal breast cancer cells. P4HTM and GATA3 co-expression is frequently associated with better patient survival. Our MET cell reprogramming model suggested that GATA3 binds to multiple loci around the P4HTM coding region including the P4HTM promoter and regulates the expression level. These results strongly suggest that P4HTM is a novel GATA3 downstream target that may be important for GATA3-mediated breast cancer cell characterization.

## Materials And Methods

### Cell culture:

T47D, MCF7, BT474, and MDA-MB-231 cells were culture in DMEM high glucose with 10% FBS.

### Western Blot:

Cells were resuspended in cold PBS and lysed with Bolt LDS Sample Buffer (Thermo Fisher Scientific). Cells were heat-inactivated at 98C° for 10 minutes and further lysed by sonication using Bioruptor UCD-200 for 10 minutes. Proteins were separated by Bolt Bis-Tris Plus Gel (Thermo Fisher Scientific). After protein transfer, the membrane was treated with the blocking buffer containing PBS-T and 5% BIO-RAD Blotting-Grade Blocker. Anti-GATA3 antibody (D13CP Cell Signaling) and Anti-P4HTM antibody (ARP57762_P050, Aviva Systems Biology) were diluted 1000 times. Images were obtained by Li-COR.

### Immunohistochemistry:

Breast cancer tissue array was purchased from TissueArray.Com (BC081116e). The assay staining protocol was developed for Dako PT Link and Autostainer Link 48 automated IHC platform. Following deparaffinization and antigen retrieval in pH6.0 buffer in the PT Link, slides were incubated with anti-P4HTM antibody (1:750) or anti-GATA3 antibody (1:1000). Thereafter, slides were incubated with a ready-to-use visualization reagent consisting of secondary antibody molecules and horseradish peroxidase molecules coupled to a dextran polymer backbone and the diaminobenzidine (DAB) chromogen, which resulted in brown precipitation of a visible reaction product localized to the antigen. Slides were counterstained with Hematoxylin, dehydrated in alcohol gradation, and mounted for microscopy. Stained slides were then scanned at 40X on the NanoZoomer Digital scanner. Slides were analyzed by NDPview2 (HAMAMATSU).

### Clinical data analysis:

Kaplan-Meier Plotter (21) was used to perform patient survival analysis. Breast cancer subtype analysis and co-expression analysis were performed by cBioportal (22, 23).

### Genomics data:

The genomics data shown in Figure 4 were obtained from the previously published data GSE72141 (17). Bigwig files and RNA-seq read counts were downloaded from this GEO data set.

## Results

### P4HTM expression is strongly correlated with GATA3

**Figure 1:**
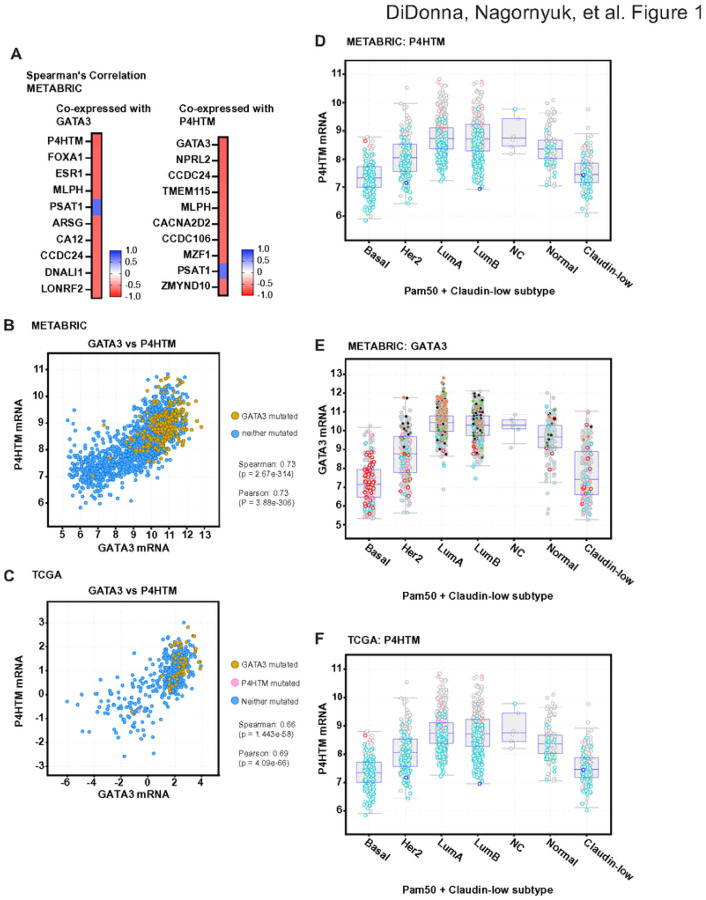
P4HTM and GATA3 co-expression in breast cancer. **(A)** Co-expression analysis was performed based on GATA3 or P4HTM expression level. Gene expression data from the METABRIC cohort was used for cBioportal co-expression analysis. Top 10 genes are listed. **(B)** Scatter plot shows GATA3 and P4HTM expression levels in the METABRIC data. **(C)** Scatter plot shows GATA3 and P4HTM expression levels in the TCGA data. **(D)** P4HTM expression in each breast cancer subtype. The METABRIC data was used. **(E)**Box plots show GATA3 expression in each breast cancer subtype (METABRIC data). **(F)**Box plots show P4HTM subtype specific expression in the TCGA cohort.

To identify novel GATA3 downstream targets that are important for breast cancer pathology, we first looked at clinical gene expression data. In the largest breast cancer cohort METABRIC data (N = 1866), P4HTM was the most highly co-expressed gene (Fig. 1A). The well-known co-factors, FOXA1 and ER-a (ESR1) were the 2nd and 3rd co-expressed genes in this cohort (Fig. 1A). We also confirmed the co-expression of GATA3 and P4HTM by looking at the co-expression data based on the P4HTM expression (Fig. 1A, right panel), and GATA3 was the most highly correlated gene once again. In the METABRIC cohort, 22% of the cases carry certain types of GATA3 mutations (including chromosome amplification or deletion, indel or missense mutations), but the GATA3 mutation status doesn’t appear to have a large impact on this co-expression (Fig. 1B). Finally, we confirmed the GATA3-P4HTM co-expression in a different data cohort. The TCGA data (N = 818) showed a strong correlation (Spearman correlation: 0.66, Pearson correlation: 0.69) between GATA3 and P4HTM expression levels (Fig. 1C).

P4HTM is a prolyl 4-hydroxylase (P4H) that catalyzes the prolyl-4 hydroxylation of proline residues of hypoxia-induced transcription factor alpha (HIF1 a). This hydroxylation is important for the down-regulation of HIF1 proteins under normoxia condition. However, the function of P4HTM in breast cancer and its relationship to GATA3 remain unknown. Thus, we decided to focus on P4HTM in breast cancer, particularly the functional interaction between P4HTM and GATA3.

Since GATA3 expression levels are known to be breast cancer subtype specific, we looked at the expression levels of P4HTM in each subtype. The expression pattern of P4HTM mirrors that of GATA3 (Fig. 1D – 1E). The highest expression was observed in Luminal A, followed by Luminal B subtype. Basal breast tumors have the lowest expression of P4HTM. This expression pattern was conserved across the different breast cancer cohorts (Fig. 1F). Collectively, transcriptome data from large patient cohorts suggested that P4HTM is a putative downstream target of GATA3 and, like GATA3, could serve as a reliable biomarker for breast cancer.

### P4htm Expression Level Is Associated With Breast Cancer Patient Prognosis

**Figure 2:**
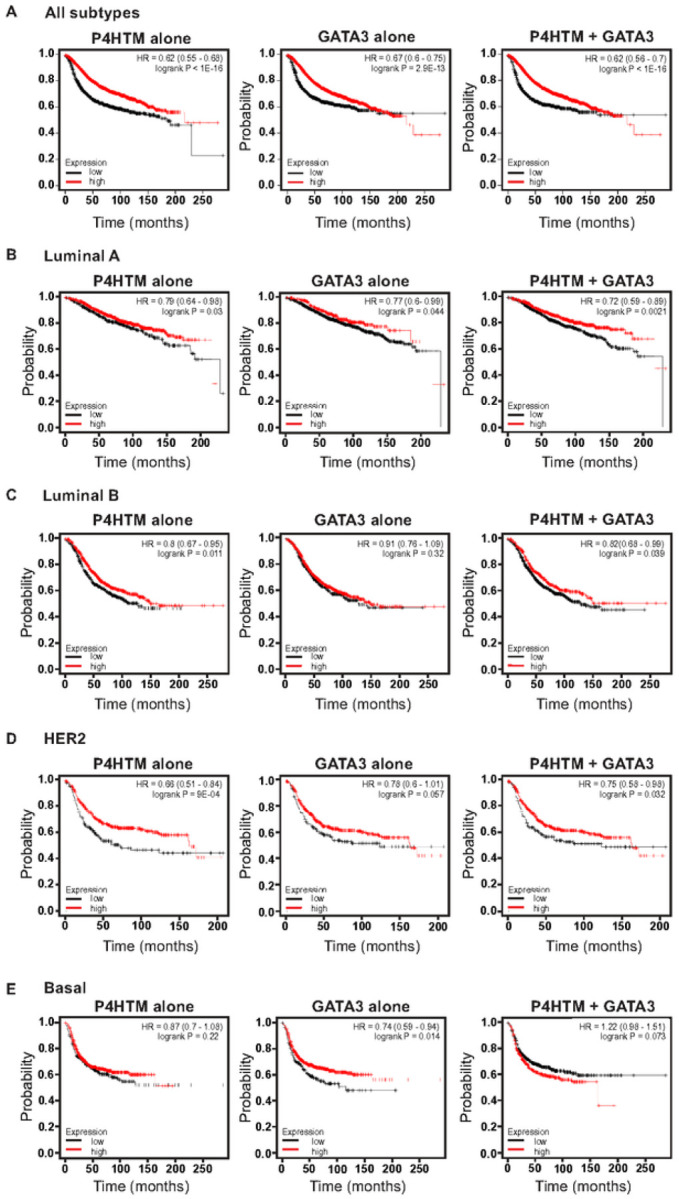
P4HTM expression levels are associated with patient survival. Patient survival analysis based on P4HTM and GATA3 expression. Kaplan-meier curves were generated by Kaplan-Meier Plotter (21). Survival analysis was performed in all subtypes (A), Luminal A (B), Luminal B (C), HER2 (D), or Basal (E) subtype. Hazard ratios (HR) and log rank p value are indicated in each panel.

Because GATA3 expression levels are associated with patient prognosis, we looked at patient survival based on P4HTM expression levels. Kaplan-Meier Plotter (21) was used for the survival analysis. Similar to GATA3, higher P4HTM expression is frequently associated with better patient survival (Fig. 2A). With the exception of basal subtype, P4HTM expression levels were positively correlated with patient survival probability (Fig. 2B – 2E). GATA3 and P4HTM differences were observed in luminal B, HER2, and basal subtypes (Fig. 2C – 2E). While lower GATA3 expression was associated with poorer patient survival, P4HTM expression levels in luminal B and HER2 cases were significantly correlated with patient survival rates. When both GATA3 and P4HTM expression levels were considered in the luminal A subtype, log rand p value became lower than individual survival analysis. These results suggest that P4HTM mRNA levels can serve as a prognostic marker, and consideration of both GATA3 and P4HTM expression can be beneficial in some subtype cases.

### P4htm Protein Expression Has Similar Pattern To Gata3

**Figure 3:**
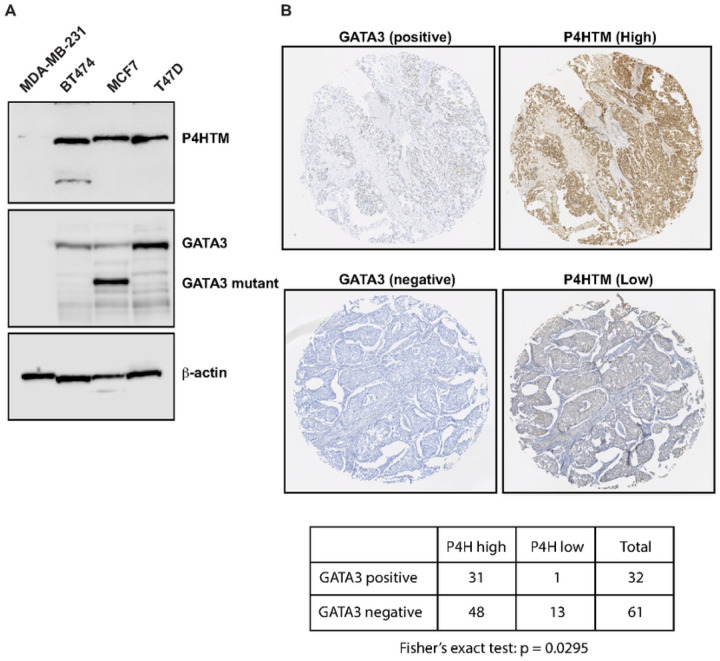
P4HTM protein expression in breast cancer cell lines and patient tumors. **(A)** P4HTM expression in breast cancer cell lines. Western blot was performed in MDA-MB-231 (basal), BT474 (HER2), MCF7 (luminal), and T47D (luminal) cells. The results for GATA3 and b-actin are also shown. (B) Representative images of immunohistochemical staining of P4HTM and GATA3 in the breast cancer tissue array. To examine the correlation between P4HTM and GATA3 staining levels, Fisher’s exact test was performed.

Although transcription levels are significantly correlated between GATA3 and P4HTM, protein expression levels may differ. To confirm the co-expression and functional network between these genes at the protein level, we first looked at P4HTM protein levels by western blot. In the widely used breast cell lines, P4HTM was observed in luminal (MCF7 and T47D) and HER2 (BT474) subtype cell lines but not in the MDA-MB-231 basal breast cancer cell line (Fig. 3A). To expand this data, immunohistochemistry for P4HTM and GATA3 was performed using a breast cancer tissue array. In this panel (93 tissues), when P4HTM staining levels were weak, breast tumors were mostly GATA3 negative (13 out of 14 cases, p = 0.0295) (Fig. 3B). Therefore, co-expression was also confirmed at the protein level.

### Gata3 Activates P4htm Gene Expression Via Chromatin Binding

To test if P4HTM is a direct downstream targe of GATA3 in breast cancer cells, we looked at the GATA3 genomic distribution at the P4HTM locus. We previously established the GATA3-induced mesenchymal-to-epithelial transition (MET) model in MDA-MB-231 cells. GATA3 expression is epigenetically silenced in MDA-MB-231 cells, and GATA3 protein expression is undetectable by western blot (Fig. 3A). Ectopic expression of GATA3 induces MET, and GATA3-expressed MDA-MB-231 cells show epithelial phenotypes such as slower cell migration and tumor growth, as well as becoming less metastatic. Since these phenotypic changes are induced by a single gene overexpression, and GATA3 well-known co-factors, FOXA1 and ER-a, are not expressed in this cell line, this system is useful for identifying a GATA3 downstream target.

**Figure 4:**
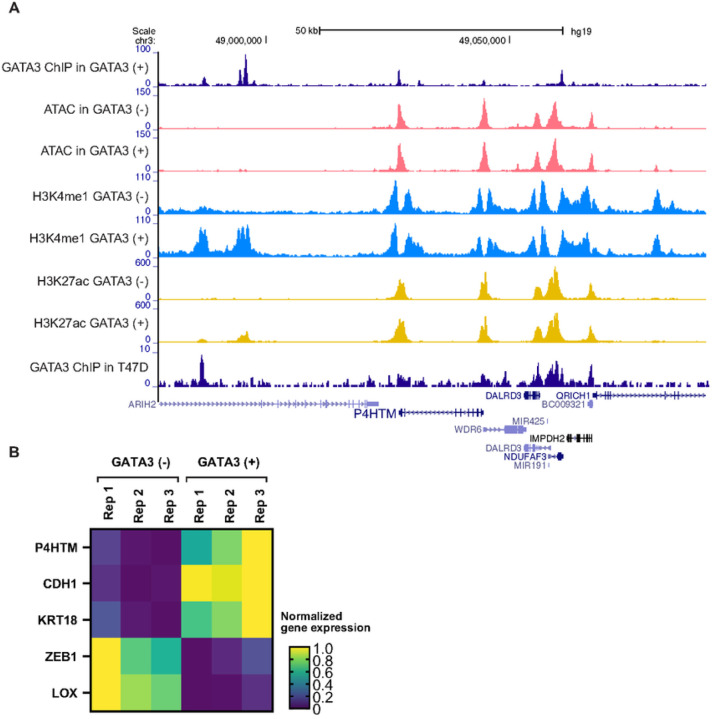
GATA3 regulates P4HTM expression in MDA-MB-231 cells. **(A)** Genome browser tracks show GATA3 ChIP-seq, ATAC-seq, active enhancer marks (H3Kme1, H3K27ac) at the P4HTM coding region. Genomics data were previously generated (17). GATA3 (−) or (+) indicates the absence or presence of GATA3 expression in MDA-MB-231 cells. **(B)** P4HTM expression in the control (GATA3 negative) or GATA3-expressed MDA-MB-231 cells. Epithelial markers (CDH1 and KRT18) and mesenchymal markers (ZEB1 and LOX) are also shown.

GATA3 ChlP-seq data showed multiple binding events around the P4HTM locus, and one of the GATA3 peaks includes the P4HTM gene promoter (Fig. 4A). While chromatin accessibility was mostly conserved before (GATA3 (–)) and after (GATA3 (+)) GATA3 expression, active enhancer histone marks (H3K4me1 and H3K27ac) were increased at the upstream region of P4HTM upon GATA3 expression. A similar binding pattern was also observed in GATA3 ChlP-seq data from T47D, a GATA3 positive cell line.

These binding events were correlated with P4HTM mRNA expression. RNA-seq data from MDA-MB-231 cells showed a significant increase (log_2_ fold change = 0.67, FDR = 5.3×10^−38^) in P4HTM expression after GATA3 ectopic expression (Fig. 4B). These results clearly indicate that GATA3 can activate P4HTM expression.

## Discussion

P4HTM, a member of the P4H family, encodes an ER transmembrane domain near its N-terminus and catalyzes the hydroxylation of proline residues (24, 25). In addition to HIF1 a, collagen, fibronectin, and laminin have been identified as P4HTM-mediated proline hydroxylation substrates. (26, 27). Because these substrates are frequently involved in tumorigenesis and cancer progression, P4HTM has been linked to cancer development. In fact, P4HTM has been shown to stimulate tumor angiogenesis in Osteosarcoma cells (26), and yet the roles of P4HTM in cancer are largely unknown.

In this study, we identified that P4HTM is a novel downstream target of GATA3 in breast cancer. P4HTM and GATA3 expression levels are often tightly correlated in various breast cancer subtypes. Similar to GATA3, higher expression of P4HTM mRNA and protein is observed in luminal breast tumors. Breast tumors with high P4HTM expression are associated with better patient survival. Genomic data from GATA3-induced MET model suggests that GATA3 regulates P4HTM expression via the P4HTM gene promoter or upstream loci. RNA-seq data confirmed that up-regulation of P4HTM is dependent on GATA3 expression. All of these data strongly suggest that P4HTM is a direct target of GATA3.

Because higher expression of GATA3 is associated with better patient prognosis and GATA3 acts as a tumor suppressor in the MDA-MB-231 MET model, P4HTM might also have a tumor suppressor function in breast cancer. In head and neck squamous cell carcinoma (HNSCC), overexpression of GATA3 was observed. Under hypoxia in HNSCC cells, GATA3 interacts with HIF1 a and stabilizes HIF1 a by inhibiting ubiquitination and proteasomal degradation (28). P4HTM-mediated proline hydroxylation, on the other hand, destabilizes HIF1 protein. It may be possible that P4HTM plays a role in disrupting the functional interaction between GATA3 and HIF1, causing HIF1a degradation even in the presence of GATA3. Other prolyl 4-hydroxylases, P4HA1 and P4HA2, were shown to regulate breast cancer metastasis via collagen deposition (27). Therefore, P4HTM could have a role in tumor progression. Further studies are necessary to elucidate the mechanism by which GATA3 and P4HTM shape the properties of breast cancer.

## References

[1] Akram M, Iqbal M, Daniyal M, Khan AU. Awareness and current knowledge of breast cancer. Biol Res. 2017;50(1):33. Epub 2017/10/04. doi: 10.1186/s40659-017-0140-9. 28969709 PMC5625777

[2] Chou J, Provot S, Werb Z. GATA3 in development and cancer differentiation: cells GATA have it! J Cell Physiol. 2010;222(1):42–9. Epub 2009/10/03. doi: 10.1002/jcp.21943. 19798694 PMC2915440

[3] Kouros-Mehr H, Slorach EM, Sternlicht MD, Werb Z. GATA-3 maintains the differentiation of the luminal cell fate in the mammary gland. Cell. 2006;127(5):1041–55. Epub 2006/11/30. doi: 10.1016/j.cell.2006.09.048. 17129787 PMC2646406

[4] Takaku M, Grimm SA, Roberts JD, Chrysovergis K, Bennett BD, Myers P GATA3 zinc finger 2 mutations reprogram the breast cancer transcriptional network. Nature communications. 2018;9(1):1059. Epub 2018/03/15. doi: 10.1038/s41467-018-03478-4. PMC5849768 29535312

[5] Ciriello G, Gatza ML, Beck AH, Wilkerson MD, Rhie SK, Pastore A, Comprehensive Molecular Portraits of Invasive Lobular Breast Cancer. Cell. 2015;163(2):506–19. Epub 2015/10/10. doi: 10.1016/j.cell.2015.09.033. 26451490 PMC4603750

[6] Pereira B, Chin SF, Rueda OM, Vollan HK, Provenzano E, Bardwell HA, The somatic mutation profiles of 2,433 breast cancers refines their genomic and transcriptomic landscapes. Nature communications. 2016;7:11479. Epub 2016/05/11. doi: 10.1038/ncomms11479. employees of Illumina Inc. Nitzan Rosenfeld is the Co-Founder and Chief Scientific Officer of Inivata Ltd. Dana W.Y. Tsui has acted as a consultant for Inivata Ltd prior to her current affiliation. Michelle Pugh is an employee of Inivata Ltd. The remaining authors declare no financial interests. PMC4866047 27161491

[7] Usary J, Llaca V, Karaca G, Presswala S, Karaca M, He X, Mutation of GATA3 in human breast tumors. Oncogene. 2004;23(46):7669–78. Epub 2004/09/14. doi: 10.1038/sj.onc.1207966. 15361840

[8] Adomas AB, Grimm SA, Malone C, Takaku M, Sims JK, Wade PA. Breast tumor specific mutation in GATA3 affects physiological mechanisms regulating transcription factor turnover. BMC cancer. 2014;14:278. Epub 2014/04/25. doi: 10.1186/1471-2407-14-278. 24758297 PMC4021073

[9] Gustin JP Miller J, Farag M, Rosen DM, Thomas M, Scharpf RB, GATA3 frameshift mutation promotes tumor growth in human luminal breast cancer cells and induces transcriptional changes seen in primary GATA3 mutant breast cancers. Oncotarget. 2017;8(61):103415–27. Epub 2017/12/22. doi: 10.18632/oncotarget.21910. 29262572 PMC5732738

[10] Emmanuel N, Lofgren KA, Peterson EA, Meier DR, Jung EH, Kenny PA. Mutant GATA3 Actively Promotes the Growth of Normal and Malignant Mammary Cells. Anticancer research. 2018;38(8):4435–41. Epub 2018/08/01. doi: 10.21873/anticanres.12745. 30061207 PMC6092927

[11] Takaku M, Grimm SA, De Kumar B, Bennett BD, Wade PA. Cancer-specific mutation of GATA3 disrupts the transcriptional regulatory network governed by Estrogen Receptor alpha, FOXA1 and GATA3. Nucleic Acids Res. 2020. Epub 2020/04/02. doi: 10.1093/nar/gkaa179. PMC7229857 32232341

[12] Hruschka N, Kalisz M, Subijana M, Grana-Castro O, Del Cano-Ochoa F, Brunet LP The GATA3 X308_Splice breast cancer mutation is a hormone context-dependent oncogenic driver. Oncogene. 2020;39(32):5455–67. Epub 2020/06/27. doi: 10.1038/s41388-020-1376-3. 32587399 PMC7410826

[13] Bertucci F, Ng CKY, Patsouris A, Droin N, Piscuoglio S, Carbuccia N, Genomic characterization of metastatic breast cancers. Nature. 2019;569(7757):560–4. Epub 2019/05/24. doi: 10.1038/s41586-019-1056-z. 31118521

[14] Takaku M, Grimm SA, Wade PA. GATA3 in Breast Cancer: Tumor Suppressor or Oncogene? Gene expression. 2015;16(4):163–8. Epub 2015/12/08. doi: 10.3727/105221615x14399878166113. 26637396 PMC4758516

[15] Chou J, Lin JH, Brenot A, Kim JW, Provot S, Werb Z. GATA3 suppresses metastasis and modulates the tumour microenvironment by regulating microRNA-29b expression. Nat Cell Biol. 2013;15(2):201–13. Epub 2013/01/29. doi: 10.1038/ncb2672. 23354167 PMC3660859

[16] Yan W, Cao QJ, Arenas RB, Bentley B, Shao R. GATA3 inhibits breast cancer metastasis through the reversal of epithelial-mesenchymal transition. The Journal of biological chemistry. 2010;285(18):14042–51. Epub 2010/03/02. doi: 10.1074/jbc.M110.105262. 20189993 PMC2859565

[17] Takaku M, Grimm SA, Shimbo T, Perera L, Menafra R, Stunnenberg HG, GATA3-dependent cellular reprogramming requires activation-domain dependent recruitment of a chromatin remodeler. Genome biology. 2016;17:36. Epub 2016/02/29. doi: 10.1186/s13059-016-0897-0. 26922637 PMC4769547

[18] Theodorou V, Stark R, Menon S, Carroll JS. GATA3 acts upstream of FOXA1 in mediating ESR1 binding by shaping enhancer accessibility. Genome research. 2013;23(1):12–22. Epub 2012/11/23. doi: 10.1101/gr.139469.112. 23172872 PMC3530671

[19] Eeckhoute J, Keeton EK, Lupien M, Krum SA, Carroll JS, Brown M. Positive cross-regulatory loop ties GATA-3 to estrogen receptor alpha expression in breast cancer. Cancer Res. 2007;67(13):6477–83. Epub 2007/07/10. doi: 10.1158/0008-5472.Can-07-0746. 17616709

[20] Saotome M, Poduval DB, Nair R, Cooper M, Takaku M. GATA3 Truncation Mutants Alter EMT Related Gene Expression via Partial Motif Recognition in Luminal Breast Cancer Cells. Front Genet. 2022;13:820532. Epub 2022/02/15. doi: 10.3389/fgene.2022.820532. 35154280 PMC8831884

[21] Lanczky A, Gyorffy B. Web-Based Survival Analysis Tool Tailored for Medical Research (KMplot): Development and implementation. J Med internet Res. 2021;23(7):e27633. Epub 2021/07/27. doi: 10.2196/27633. 34309564 PMC8367126

[22] Cerami E, Gao J, Dogrusoz U, Gross BE, Sumer SO, Aksoy BA, The cBio cancer genomics portal: an open platform for exploring multidimensional cancer genomics data. Cancer Discov. 2012;2(5):401–4. Epub 2012/05/17. doi: 10.1158/2159-8290.Cd-12-0095. 22588877 PMC3956037

[23] Gao J, Aksoy BA, Dogrusoz U, Dresdner G, Gross B, Sumer SO, integrative analysis of complex cancer genomics and clinical profiles using the cBioPortal. Sci Signal. 2013;6(269):pl1. Epub 2013/04/04. doi: 10.1126/scisignal.2004088. 23550210 PMC4160307

[24] Byts N, Sharma S, Laurila J, Paudel P Miinalainen i, Ronkainen VP Transmembrane Prolyl 4-Hydroxylase is a Novel Regulator of Calcium Signaling in Astrocytes. eNeuro. 2021;8(1). Epub 2020/12/11. doi: 10.1523/eneuro.0253-20.2020. PMC7814479 33298456

[25] Myllykoski M, Sutinen A, Koski MK, Kallio JP Raasakka A, Myllyharju J, Structure of transmembrane prolyl 4-hydroxylase reveals unique organization of EF and dioxygenase domains. The Journal of biological chemistry. 2021;296:100197. Epub 2020/12/19. doi: 10.1074/jbc.RA120.016542. 33334883 PMC7948501

[26] Klotzsche-von Ameln A, Prade i, Grosser M, Kettelhake A, Rezaei M, Chavakis T, PHD4 stimulates tumor angiogenesis in osteosarcoma cells via TGF-α. Mol Cancer Res. 2013;11(11):1337–48. Epub 2013/09/21. doi: 10.1158/1541-7786.Mcr-13-0201. 24048703

[27] Gilkes DM, Chaturvedi P Bajpai S, Wong CC, Wei H, Pitcairn S, Collagen prolyl hydroxylases are essential for breast cancer metastasis. Cancer Res. 2013;73(11):3285–96. Epub 2013/03/30. doi: 10.1158/0008-5472.Can-12-3963. 23539444 PMC3674184

[28] Lin MC, Lin JJ, Hsu CL, Juan HF, Lou PJ, Huang MC. GATA3 interacts with and stabilizes HiF-1α to enhance cancer cell invasiveness. Oncogene. 2017;36(30):4243–52. Epub 2017/03/07. doi: 10.1038/onc.2017.8. 28263977 PMC5537608

